# ‘Save our NHS’: activism, information-based expertise and the ‘new times’ of the 1980s

**DOI:** 10.1080/13619462.2018.1525299

**Published:** 2018-09-25

**Authors:** Jennifer Crane

**Affiliations:** Centre for the History of Medicine, University of Warwick, Coventry, UK

**Keywords:** NHS, activism, campaigning, expertise, 1980s

## Abstract

This article examines activism in defence of the National Health Service (NHS), which emerges in the 1960s to defend local hospitals from closure. From the mid-1980s, a new form of campaigning developed, which sought to protect the Service nationally. Tracing this campaigning illuminates, first, that small groups played a significant role in negotiating political change, and in contributing to cultural change which, in turn, has become politically powerful. Second, this demonstrates that the 1980s were ‘new times’ in welfare politics, as Thatcher’s changes fostered voluntary interest in information-led expertise, and a new vision of the NHS as a significant, much valued, national institution.

## Introduction

One of the reasons we put the NHS in the [2012 Olympics Opening] show is that everyone is aware of how important the NHS is to everybody in this country.^1^

Danny Boyle

I couldn’t help but smile when we first saw the bit when the monsters came out. Are they supposed to be the managers or the politicians?^2^

NHS worker

Britain’s National Health Service (NHS) assumed a key place within the Olympic opening ceremony of 2012, directed by Danny Boyle. Following representations of the Industrial Revolution, and a comedy skit of James Bond parachuting into the Stadium with the Queen, 600 NHS staff and 1200 volunteers jived around the logos of Great Ormond Street Hospital and the NHS. The significance and popularity of the NHS was stressed by Boyle, emphasising that ‘everyone’ was aware of its importance. This message was underscored by the ceremony’s press-pack, which discussed the NHS as a uniquely post-war institution that, ‘more than anything unites our nation’.^3^ The distinctly ‘British’ nature of this celebration was articulated by newspapers, which gleefully quoted American commentators, ‘baffled by [the] NHS tribute’.^4^

Amidst this lively representation, however, lurked discontent. The ceremony dance sequence itself, blending into a celebration of children’s literature, featured puppets representing villains such as pirate Captain Hook, Lord Voldemort from the Harry Potter series and Disney’s Cruella de Vil. Discussing the ceremony, one NHS dancer, a member of staff in the Service, provocatively asked the *Guardian* whether these ‘monsters’ were perhaps intended to represent managers or politicians.^5^ Further, Boyle was quoted in the *Daily Mirror* stating that the political ‘forces’ had wanted him to cancel or to reduce the length of his NHS-themed segment.^6^ This article explains, explores and contextualises these contrasting representations: between the NHS as precious and yet under threat; as culturally valuable yet politically vulnerable; and as a symbol of ‘British values’ but also a globally recognised ‘brand’.

As the first section of this article demonstrates, from the inception of the NHS in 1948, campaigners sought to lobby to change policy and practice, for example around the treatment of specific diseases and the representation of patients. Notably, campaign groups also developed to contest specific hospital closures, conceptualised through rich descriptions of the unique architectural and atmospheric benefits of these sites. This campaigning—and the campaigning of Community Health Councils—promoted a vision whereby publics and policy interfaced about the NHS through the local level, and often through lobbying, protests, occupations and strikes. The second section of this article contends, indeed, that campaigning around the NHS changed significantly in the 1980s. It was in this decade, and in response to changes—proposed and imagined—by Margaret Thatcher, that campaign groups began to mobilise in defence of the NHS itself, arguing that this institution *as a whole* was important, yet under threat. It was also in this decade that new national campaign groups, while still very small, began to deploy new forms of expertise grounded in the provision and analysis of information, often gleaned through leaks. This was a significant shift in campaigning cultures, although continuities remained with the activism of the mid-twentieth century, notably in terms of the role played by media and the ongoing significance of locally based loyalties.

In tracing this change, this article contributes to works, for example by Pat Thane, Tanya Evans, Alex Mold, Virginia Berridge and Chris Moores, demonstrating that the study of very small voluntary organisations—much smaller than non-governmental organisations, New Social Movement Organisations or charities—can significantly enhance our thinking about contemporary British history. The groups studied in this article were often very small. One of the article’s largest case studies, London Health Emergency (LHE; 1983–present), had just three members of staff at its peak, and its annual income fluctuated between £40,000 and £70,000.^7^ Nonetheless, examination of this group, its papers and archival materials, provides significant insight into how political change, for example led by Margaret Thatcher, has been lived and felt in everyday life. The case studies of this article also provide insight into how such small voluntary groups have, through voluntary networks and media interest, reflected, negotiated, and even to an extent contributed to, political change. Indeed, looking at these small voluntary groups, and at the 1980s moment, reveals the inception of the cultural power of the NHS in recent years, as evidenced by, for example, the frenzy of the Olympics Opening Ceremony.

## ‘Save our hospitals’, 1948–1970s

Activism around the NHS has mirrored and reflected the social policies and spending priorities of successive governments. In the period before and immediately following the introduction of the NHS, political debate assessed whether the Service should be managed on a local or a national level, and how state medicine would interact with, reduce or manage the budgets and working lives of doctors.^8^ Accordingly, in this period activist commentary on the health service was dominated by professional organisations, such as the British Medical Association, Socialist Medical Association and the Fellowship for Freedom in Medicine, who campaigned for and against the provision of nationalised medicine. John Stewart has described how the Socialist Medical Association campaigned for healthcare for all at the point of access from 1930.^9^ Conversely, Andrew Seaton has analysed the Fellowship for Freedom in Medicine, founded in 1948, which sought to present the NHS as an ‘economically dangerous bureaucratic machine that crushed medical independence and risked pushing the country towards dictatorship’.^10^

In the early-to-mid 1950s, such thinking—fundamentally agitating for or against the existence of the NHS itself—died down as the NHS entered an ‘initial phase of consolidation’.^11^ Social policy was more interested in housing and education than health, and there was little campaigning momentum around either defending or attacking the NHS itself. Nonetheless certain inquiries, such as the *Guillebaud Report* (1956), began to discuss the long-term financial efficiency and regional distribution of the service.^12^ Activism continued, but primarily in terms of seeking to influence how the NHS functioned and definitions of ‘health’. Activism by families, communities and individuals—continuing work from the nineteenth and twentieth centuries—sought to provide services for, and direct funding and attention towards, disability, diabetes, breast cancer, rickets, sickle cell anaemia, AIDS/HIV, drug use, birth, reproductive health, and maternal and paternal roles, for example.^13^

In the contexts of an expanding economy, a ‘technocratic moment’, and growing political interest in planning for health in the late 1950s and early 1960s, spending on hospital building grew from £10 million in 1956–1957 to £31 million in 1961–1962.^14^ Enoch Powell, Minister of Health from 1960 to 1963, was interested in this work, and in tying hospital building to a perceived ‘rationalisation’ of the NHS, including the integration of primary, secondary, community and social services. The *Hospital Plan for England and Wales* (1962) proposed a ten-year plan, looking to reform the provision of hospitals, previously determined by the sizes of the local and voluntary hospitals nationalised at the inception of the NHS. Instead, the *Plan* recommended the creation of a new system of District General Hospitals, which would be distributed equally across Britain and operate with similar size populations, and providing all types of care from the majority of specialities.^15^

This plan was not totally fulfilled, given limitations to public expenditure and to the capacity of the building industry.^16^ Nonetheless, this plan signified an ongoing debate about the relationships between resources and regions in the NHS. Policymakers throughout the 1960s and 1970s sought to resolve this debate by constructing new formulae, notably the Crossman formula and, later, through the Resource Allocation Working Party. These formulae gave varying weight to need, teaching and existing provision.^17^ Martin Gorsky has argued that the Crossman formula in particular, devised in the late 1960s and taken up by Keith Joseph in the early 1970s, meant, ‘the beginnings of an academic debate were heard, a policy discourse gathered pace, and a policy window started to open’.^18^ The first major reorganisation of the NHS in 1974 created a new system of ‘area’ and ‘district’ health authorities, and marked an era of ongoing debate about the shape and structure of the NHS, its priorities and inequalities.^19^

Significantly, and interacting with these phenomena, this late 1960s and early 1970s moment also represented a new era of NHS activism, within which campaigners, communities, and NHS staff sought to influence and contest the redistribution of NHS resources, particularly as economic pressures continued through the 1970s. NHS staff, in this decade, undertook their first industrial action about pay, spending and safety.^20^ The *NHS Reorganisation Act* of 1973 established Community Health Councils, which sought to consult with local publics and to lobby, on their behalf, to reshape health provisions.^21^ In another key site of activism, many small, local campaign groups emerged in response to local cuts, including the New End Hospital Defence Committee, Save the West London Hospital Campaign, and Bethlem and Maudsley Action Committee. These campaigns varied in terms of their size and organisation. One well-documented campaign was that to save Bethnal Green Hospital, after the Department of Health planned to convert it into a geriatric-only unit (closing its Acute and Casualty beds). The Save Bethnal Green Hospital Campaign was run by a seasoned activist, the radical General Practitioner (GP) David Widgery, who had worked at the hospital and who found further support from 102 other local GPs.^22^

New sites of activism, therefore, mirrored and reflected changes in NHS policy from its inception. In the 1960s and 1970s, NHS campaigning was primarily based on locale. Community Health Councils were designed to serve populations of 86,000 to 530,000 people, and to represent local needs.^23^ Anti-closure campaigns often emphasised the unique and special nature of their specific hospitals, relating sentiment about particular hospitals to their architecture and histories. The campaign against the closure of the Elizabeth Garrett Anderson Hospital, for instance, one of only two hospitals at this time staffed only by women, and serving only women, emphasised that its ‘smallness’ created a ‘caring’ and ‘friendly and intimate atmosphere’. The design of the hospital, described as ‘light and bright and airy’, was seen to shape this atmosphere, and indeed was negatively compared to ‘the often oppressive regimental atmosphere in big hospitals’.^24^

Likewise, the Hands Off Rhydlafar campaign, led by Cardiff Trades Union Council, described their hospital as ‘beautiful & modern’ and accused the local Area Health Authority of wanting to ‘vandalise the place and sell the site’.^25^ Contemporary newspaper coverage similarly personified hospitals facing closure, discussing how they faced their own individual ‘survival battle[s]’ or were ‘facing death’.^26^ Notions of local history were embedded within campaigning, and newspaper reports and campaign banners emphasised that hospitals had a ‘great history’, were ‘founded 232 years ago’ and had provided ‘100 years of medical service in the area’.^27^ Early campaigns were thus deeply tied to a specific sense of space, time and place, rather than to a vision of the ‘NHS’ more broadly. Different aspects of specific hospitals underpinned their perceived utility and value, whether around the light, airiness, history, modernity or beauty of the sites. For women’s hospitals, specifically, ideas about ‘cosiness’ and ‘friendliness’ held gendered resonances.

The key tactics of anti-closure campaign groups likewise centred on the specific spaces of individual hospitals, and focused on work-ins, occupations, protest marches, and, in a campaign in East London in 1973, an attempt by staff to sacrifice their wages to keep a hospital open.^28^ These forms of activism were primarily conducted by staff, but also fortified by local communities and often by previous patients. Protesting women and families, ‘parents, schoolchildren, expectant mothers and babies’, tended to garner the most press interest.^29^ In 1971, a protest against the closure of Queen Charlotte’s Maternity Hospital in West London, the *Observer* reported, involved a ‘mass lobby of women’, ‘many of whom have had their babies at the hospital’, and who returned ‘armed with babies, banners’, and a petition.^30^ The *Guardian* and *Observer* were particularly keen to document such activism, contributing to new interest in the political lives of women and mothers.^31^ Recognising this, and paying tribute to the role of the media in this moment, in February 1965 a campaign group which had ‘saved’ Queen Mary’s Hospital for Children, in Carshalton, thanked the newspaper for providing ‘invaluable square inches of space’.^32^

Demonstrating belief that these active forms of protest were the most effective campaigning tactics in this period, the artists Loraine Leeson and Peter Dunn, who supported the Bethnal Green campaign, produced posters which argued that, ‘it is ultimately organised mass support which can wield most power’, and the group documented attendance numbers at public meetings and marches.^33^ Dr Jean Lawrie, Chairman of the Hospital Action Committee against closures at the Elizabeth Garrett Anderson Hospital, likewise positioned these tactics as a product of their time, arguing in 1977 that, ‘I’m told that demonstrations have to be made these days to show anyone that you really *mean* what you say’.^34^ While therefore seen as a contemporary phenomenon, these tactics were not universally popular with locals or hospital staff. Indeed, the *Guardian* stated in 1977 that some staff at the Garrett Anderson hospital found the ‘militancy’ of picketing ‘curiously embarrassing’.^35^ This demonstrated a level of unease even among left-wing press at this time with industrial action, particularly around a women’s hospital, and the *Guardian* defensively further stated that protestors were ‘not wild women’s libbers’.^36^ Other types of campaigning organisation pursued alternative modes of activism, though still grounded on the local level: Community Health Councils, for example, encouraged and facilitated letter-writing, petitions and meetings directed towards local MPs or Regional Health Authorities.

Overall, therefore, early campaigning cultures around the NHS—formed from the inception of the Service but particularly visible from the 1960s and 1970s—were primarily grounded in a vision of local action, and particularly focused on local hospital provision, continuing long-term interest in hospitals as primary employers and as a foci for local philanthropy.^37^ The national movement in defence of ‘the NHS’, traced in the following sections of this article, had to be constructed, forged and fought for, and did not emerge organically. While public attachment to the NHS, as expressed and negotiated through voluntary action, was mediated locally in these decades, at times public feelings did nonetheless become politically powerful on a national level. The campaign around the Elizabeth Garrett Anderson Hospital was significant in this regard, and began to accrue countrywide support, particularly as the hospital served a sweeping population of women, and thus represented women’s interests more broadly, as well as the women of London.^38^ Indeed, having provided funding for this hospital when coming to office in 1979, Margaret Thatcher told the Conservative Party conference in 1986 that her government had ‘saved’ this hospital and could thus be trusted with the broader health service.^39^ This statement, while political rhetoric, was also symbolic of the ways in which small-level organised protests could, by the 1980s, contribute to and reshape political debates—albeit not always in the ways that protesters had initially imagined.

## ‘Save our NHS’, 1983–1990s

The nature of NHS activism changed significantly from the 1980s. Reshaped by left-wing revival under Thatcher, campaigning moved to focus on the NHS, rather than on local hospitals, and also emphasised the significance of ‘evidence’ and ‘information’ in welfare politics. These changes—and the role of the voluntary sector in promoting them—are difficult to trace, given the transient nature of many voluntary groups in this area. However, the changes are made visible through a close case study of the organisation LHE. LHE was founded in 1983, and was one of the few NHS-related campaign organisations which continued to act, without interruption or dissipation, throughout the 1980s and 1990s. The organisation has left a significant archive at the Modern Records Centre at the University of Warwick, enabling a unique opportunity to trace its work and, through doing so, to understand broader networks of NHS activism.

LHE was an important contributor to broader debates about the NHS in the late twentieth century. The organisation provided consultancy work, regular media comment and, by 1985, had 225 affiliates, including national, regional and local branches of trade unions, notably the National Union of Public Employees and the Confederation of Health Service Employees; trades councils; Community Health Councils; Labour Parties; other NHS-related campaigns; community groups; and pensioner groups.^40^ By 2005, the organisation remained significant, and its leaders co-founded the large-scale campaign group ‘Keep Our NHS Public’, in collaboration with the NHS Support Federation and the NHS Consultants Association.

LHE was a relatively small organisation: its magazine, *Health Emergency*, had a print run of 14,000 in 1984 and 16,000 in 1988.^41^ Nonetheless, this organisation embodied, and was significant within, broader shifts in voluntary cultures around the NHS in the 1980s. Indeed, LHE emerged while Thatcher’s administrations made significant changes to NHS policy: slowing the growth of real expenditure; introducing a more ‘thrusting’ management style; encouraging private sector involvement; and, from 1989, introducing an internal market, bringing in a divide between ‘purchasers’ and ‘providers’.^42^ While historians debate the extent to which Thatcher’s policies were driven by ideology or pragmatism, and their significance, radicalism and reach, certainly the Thatcher administrations developed a ‘confrontational rhetoric’ around the NHS and welfare.^43^

This confrontational rhetoric drove the formation of new protest organisations and solidarities.^44^ LHE was such a new group; founded by the Greater London Council (GLC) in 1983, explicitly as Ken Livingstone, the GLC leader, sought to bolster voluntary organisations, Stephen Brooke has argued, as a ‘defiant bloom set against neo-liberalism'.^45^ LHE was indeed initially driven by a coalition of ‘left-wing’ interests. The three members of staff first employed by the GLC at LHE were from backgrounds with broadly ‘left-wing’ organisations and trade unions.^46^ After the GLC was abolished in 1986, LHE first gained its funding from a group of left-wing local authorities, including Camden, Ealing and Greenwich.^47^

LHE thus emerged from a context in which Thatcher’s reforms were perceived and presented, by activists and Labour politicians alike, as a fundamental threat to ‘the basic idea of the NHS’.^48^ Recognising this, John Lister, a leader at LHE, reflected in 1988 that perhaps Thatcher’s ‘only contribution to health issues’ was ‘bring[ing] the NHS firmly back into the political arena’.^49^ In this context, the visual representation of Thatcher became an important mobilising symbol and clear target for NHS activism. Local protest groups displayed giant puppets of Margaret Thatcher and ‘the Faceless Bureaucrat’ wielding axes at events.^50^ Staff rallies, such as one ‘in pouring rain!’ in Oxford in 1988, included banners with slogans such as, ‘Maggie Makes Us Sick’.^51^ Further making Thatcher, specifically, the proxy for all NHS policy in this era, LHE newsletters likewise discussed Thatcher’s use of a private hospital for an eye operation, ‘carried out with NHS equipment borrowed from a local hospital’; described spending reductions as ‘Thatcher’s cuts’; and allied discussions of health strikes with those in the mining industry.^52^ Seeking to form new solidarities across professional lines and against ‘Thatcherism’, in June 1984 *Health Emergency* reported that the ‘struggle to save the NHS and to save the coal industry’ was ‘a single fight’ acting ‘against this government and its monetarist policies’.^53^

Therefore, a new strand of NHS activism emerged in response to Thatcher, embodied by LHE. This new activism was in part driven by a rejection of Thatcher’s policies, and explicitly made Thatcher a symbol of protest. This new association between NHS activism and left-wing politics in part overturned the focus of previous 1970s anti-closure groups on working pragmatically with local politicians, crosscutting national political agendas. While this new politics was closely linked to left-wing politics, however, it was not solely defined by it. Indeed, two further significant components emerged in this new form of NHS activism: a focus on national cultures, rather than local ones, and an interest in using information as expertise. These new foci conflicted with and began to replace earlier voluntary interests in local areas, and in active forms of protest such as sit-ins and strikes. Further, these new foci also reflected and contributed to changing visions of the NHS, activism and expertise in the 1980s Britain.

## National interests

The new NHS activism of the 1980s was increasingly focused on constructing a vision of the NHS, rather than campaigning on a local level, as through the 1940s to the 1970s. Again, this shift was exemplified by the work of LHE. While initially only founded to defend London, LHE’s mandate was broader than the defence of individual hospitals. Representatives from the group emphasised that it became determined ‘to reflect national rather than simply London issues’, to construct a *national* movement to defend the whole NHS and to lead a ‘fight for our NHS’ or to ‘Stand up for the NHS’, as a whole.^54^ Within the group’s material culture, the vision of a national Service—‘our NHS’—also became significant. In response to the White Paper, *Working for Patients*, LHE launched a ‘Hands Off Our NHS’ campaign in February 1989, producing badges, balloons, car stickers and t-shirts bearing this slogan.^55^ Through the creation of material culture, group organisers sought to make support for the NHS, as a whole, significant in everyday life and in the formation of individual and collective identity. An imagined community of campaigners was further fostered through LHE publications as newsletters stated, for example, ‘It’s up to us to defend our NHS!’, and pictured placards compelling people to ‘Save *Your* Health Service’.^56^ This shifting culture of activism—from local to national areas—was also evidenced as LHE staff began to focus their time on representing and supporting groups from across the country. Staff toured the nation, visiting meetings, and in the media they highlighted cases and cuts from across Britain. Indeed, *Health Emergency* regularly called on its readers to defend all areas of the country targeted by cuts writing, for example, that, ‘It’s London today, and the rest of the country tomorrow’, or that, ‘If it has been allowed to happen to Queen Mary’s University Hospital in Roehampton it could happen to *your* hospital, too.’^57^

LHE was representing and seeking to drive a broader movement, and many other campaigners were also shifting their focus beyond local hospitals in the 1980s. At the turn of the 1980s, the campaign group Fightback praised ‘active local groups’ for their ‘bold initiatives and imaginative tactics’, but also argued that ‘we are still far too weak to stop the cuts nationally’.^58^ NHS staff and trade unions were also beginning to fear that the NHS as a whole was under threat: by the late 1980s, protest banners stated, ‘RIP NHS’, as well as referring to individual hospital closures.^59^ In July 1990, the LHE newsletter carried an advertisement from a health union, the Confederation of Health Service Employees, calling ‘For a Better National Health Service’ and attacking ‘the Government’s Plans to Fragment It’.^60^ An indication of the longer-term success and significance of this ‘save the NHS’ message, new to the 1980s, was that Keep Our NHS Public, when founded in 2005, looked to unite broad factions of ‘the public’ in favour of the NHS on a national level. Supporters insisted that Keep Our NHS Public had ‘taken off as a nation-wide and broad campaign’, rather than as a series of local efforts alone.^61^

Notably, shifting interest from campaigning around the NHS as a ‘local’ to a ‘national’ service did not merely signal a shift in geographical focus. Rather, by changing focus towards the ‘national’, groups such as LHE also sought to broaden the focus of campaigns from hospitals specifically towards the whole of the NHS, looking to join interest in ‘the cause and the cure of illness’ and to encompass analysis of health behaviours, community care, GPs and services such as physiotherapy.^62^ The shift to the ‘national’ also led calls to look more broadly at inequality and discrimination in the Service. This shift built on the longer term work of the Politics of Health Group and the Socialist Medical Association, and also reflected the focus of new research, for example by the Working Group on Inequalities, which produced the *Black Report* (1980).^63^ Indeed, critiquing campaigning which focused on hospital closures alone, rather than structural change, a journal of the Socialist Medical Association, *Socialism & Health*, argued in its October 1979 edition that the campaign to save the Elizabeth Garrett Anderson Hospital was, or should be, ‘not just a struggle to prevent a hospital closure but also the demand for a better women’s health service’.^64^ By the 1980s, specific campaigns against the closure of women’s hospitals were joined by those, led by LHE, for the provision of cervical screening and against the gendered divisions of community care.^65^ LHE also sought to direct attention towards the ‘racist attitudes of the Health Authorities and the medical hierarchy’, for example in lack of attention paid to sickle cell anaemia; an inherited disorder primarily affecting people with an African or Caribbean family background.^66^

These types of shift—from local-based campaigning around individual hospitals towards national campaigning around an equitable health service—underscored the new 1980s activist visions of the NHS as embodying a certain set of *values*, as well as a system of healthcare. Activist critique would then ardently defend the perceived values of the NHS, while critiquing how, because of reforms and funding limitations, it operated in practice. A recent survey of the views of 175 self-identified NHS campaigners showed the ways in which visions of NHS, nation and value have continued to be co-constructed. In response to a question about why people became campaigners, participants referenced their desire ‘to be part of a national campaign to save the NHS’, which was the ‘UK’s real crown jewels’, ‘the pride of Britain’ and ‘the best health service in the world’. No participants stated that they had joined to save their local hospitals specifically, but rather that they feared that the NHS as a whole was under attack by ‘ideologies’, ‘Tories’, ‘privatisation’ and ‘neoliberalism’.^67^ This construction paid less attention to the significance of individual hospitals, losing the campaigning interest from the 1960s and 1970s in whether local sites were ‘small’, ‘caring’, ‘friendly’, ‘modern’ or ‘beautiful’.

While the work of 1980s campaigners therefore fostered a vision of the NHS as a national service—a vision which remains significant today—strong loyalties at the local level also proved challenging for campaigners’ work. This was typified when LHE’s attempt to establish a ‘National Health Emergency’ group floundered, or at least left few archival traces.^68^ Lister later reflected in 2016 that people may relate more strongly to local and immediate, than to general, concerns and issues.^69^ Reflecting and contributing to these tensions, at times the LHE’s campaigning remained somewhat London-centric. The rhetoric around the early work of LHE paralleled that of earlier local groups, in that local supporters repeatedly underscored the uniqueness of London’s hospitals and healthcare needs. In 1984, Frank Dobson, the Labour MP for Holborn and St Pancras—and future Secretary of State for Health—testified that: ‘there is a need for a London-wide organisation to get together the figures, to present a London-wide picture’, as well as to ‘help all the local campaigns learn from one another’s successes and mistakes’.^70^

Tensions between the local and the national were magnified in the early 1990s as two reports—by the King’s Fund and the Tomlinson Inquiry (the latter a public inquiry established by Health Secretary William Waldegrave)—lobbied for the closure of several of London’s hospitals, to be replaced by increased primary and community care provision.^71^ LHE publications argued that it was not in fact London but rather Liverpool, Manchester or Newcastle whose hospitals were over-funded.^72^ Further suggesting the significance of ongoing regional loyalties, the group also attacked Bernard Tomlinson, Chair of the Tomlinson Inquiry and of the Northern Regional Health Authority, as ‘[Health Secretary Virginia] Bottomley’s man from the Northern Region’.^73^ Local ties have continued to motivate local activism in recent years. For example, in 2001 Dr Richard Taylor was elected to Parliament as part of the ‘Save Kidderminster Hospital’ campaign, and local campaigning was critical in preventing the closure of the Accident and Emergency ward in Lewisham Hospital in 2013.^74^

Earlier NHS history was characterised by anti-NHS activism—documented by Seaton—and by campaigning in defence of local services or around specific diseases. Nationally focused activism in defence of the NHS was new to the 1980s. It did not emerge organically, but rather was in part forged by the active work of LHE and other campaign groups. NHS activism had changed in this decade but, notably, campaigners also faced the same tensions as were implicit in NHS policy: between focusing on the national and the local, and between centralisation and devolution of resources, funding and responsibility.^75^

## Information-based campaigning

Another facet of NHS campaigning which was new to the 1980s was the emphasis on ‘information-led expertise’, to be provided not only by large non-governmental organisations but also by small voluntary groups. LHE again embodied this change and consciously enacted information-based activism, regularly seeking to emphasise that their work was, first, ‘an accurate picture of grassroots opinion’ and, second, based on analysis of information and ‘facts’.^76^ The second part of this formulation was key to LHE’s self-framing, and staff emphasised that they offered ‘neutral’ information, in contrast to the ‘political’ or ‘propagandist’ narratives, or even ‘conspiracy theories’, provided by other organisations.^77^

Despite being relatively small in size, LHE’s annual report of 1985 declared that it was a ‘prime source of information, advice, experience and contacts among health unions, campaigns in London and elsewhere, and among journalists covering the NHS’.^78^ LHE’s 1987 annual report proclaimed that the group had ‘continued to establish its reputation as a major authority on the state of London’s NHS’; been approached as a ‘primary source of information’; and built up ‘an essential information base on London’s NHS’.^79^ Notably, newspaper coverage tended to accept this conceptualisation of the LHE’s role, referring to the group variously as a ‘monitoring organisation’, ‘watchdog organisation’ and ‘patients’ or ‘hospital’ ‘watchdog’, though also at times as ‘an organisation campaigning against cuts’ or a ‘pressure group’.^80^ In part, therefore, the alignment of LHE with ‘information’ served as a defence from accusations of acting ‘politically’, and functioned to resist a singular association of defence of the NHS with left-wing politics.

This new focus on providing information transcended LHE alone, and built on earlier interest in research and sociology by, for example, the campaign groups Shelter and the Child Poverty Action Group, working in the 1960s and 1970s.^81^ The 1980s interest in information from NHS activists, specifically, was distinct because it was constructed in a period when the NHS itself was also increasingly mandated to provide ‘information’. In the 1970s, Chris Ham has argued, the effectiveness of Community Health Councils was hindered by ‘difficulty in acquiring information’, to put up counter proposals against suggested hospital closures.^82^ By the 1980s and particularly through the 1990s, by contrast, the NHS began to provide performance indicators, patients’ charters and rights guides, waiting lists and increased access to patient records; in part responding to campaigns from patient groups.^83^ At the same time, other voluntary organisations were also seeking to create and critique ‘information’ about the NHS in new ways. The campaign group NHS Unlimited, established in April 1981 in the House of Commons, aimed to promote ‘a well-informed defence of the NHS’ by spreading information, particularly about the costs of private health insurance.^84^ The Politics of Health Group established a ‘Women’s Health Information Centre Collective’, founding an accessible library for women to educate themselves about their bodies and health.^85^

While Hilton, McKay, Crowson, and Mouhot have drawn valuable attention to the politics of expertise visible in large non-government organisations of the post-war period, it is significant also that even these smaller groups sought to manifest information-based expertise.^86^ LHE was significant in this process. Notably, the group used information differently to large non-governmental organisations. Its focus—and that of NHS Unlimited and the Women’s Health Information Centre—was around providing information to broader publics as a tool of empowerment, as well as in informing ‘expert’ debate between professions or policy. Most significantly, perhaps, and in part because of LHE’s smaller size, the organisation also differed from large non-governmental organisations in terms of receiving and disseminating ‘leaks’, and in making particularly sustained and brutal criticism of the ‘facts’ provided by other groupings. At the same time, LHE also replicated the work of non-governmental organisations, particularly in constructing analysis and ‘facts’ about health policy.

### Leaks

The first key use of information at LHE was ‘leaks’, underpinned by the group’s belief that successive governments—not only the Thatcher governments—held ‘hidden agenda[s]’, which were being ‘kept under wraps for fear of the electoral consequences’.^87^ With this in mind, the group leaked numerous documents, including plans for London’s hospitals produced by the London Implementation Group and the Department of Health in the 1990s. LHE also leaked letters used to brief NHS and government staff, such as, in 1985, a document for managers of social security offices, which advised that they chose words ‘very carefully indeed’ when communicating with the public and avoided any perceived criticism of government policy.^88^ A range of sources provided these documents, including a hospital cleaner and a worker at a Regional Health Authority, as well as Parliamentary officials.^89^ LHE usually provided their leaks to all newspapers, regardless of their political leanings or standing, although at times they excluded the *Daily Mail*, in response to hostile coverage.^90^

Previous campaign groups around the NHS had not found nor leaked government documents in this manner; this was new. The way in which LHE shared information as widely as possible, and with journalists working for national publications, contrasted with the sharing of information by earlier campaigns against specific hospital closures, who tended to provide information only to campaigners and members of the public in their local areas, and who had also focused on physical, rather than written, forms of political participation. LHE was able to disseminate information broadly because they had information of interest to national media, and because the group had members of staff dedicated to, and experienced in, working with press officials. Notably, LHE’s ability to receive and use leaks also reflected changing management styles within Thatcher’s civil service. Staff at LHE contended that they had begun to leak documents in response to a context in the 1980s and 1990s in which health authorities were displaying ‘increasing secrecy’.^91^ David Vincent has demonstrated that obtaining leaked materials may have become easier at this time, as disgruntled Parliamentary officials were increasingly willing to share documents.^92^ Leaking was also a result of the increasing number of NHS reorganisations, which meant that more documents were produced, copied and shared.

LHE thus was manifesting a politics of information-led expertise akin to large non-governmental organisations: the group was becoming influential in health policy debates and also acting ‘professionally’, mediating between public and policy communities. At the same time, LHE’s expertise was also different to that of large-scale voluntary organisations. In part because this group was small, and because it was relatively ‘radical’, it was able to receive and leak confidential information without significant legal or reputational damage.

### Constructing facts

As well as sharing leaked documents, LHE staff also sought to construct facts; disseminating reports which they themselves had created. The group regularly produced ‘Fact Sheets’ for the public, which were sold for just 45 pence (or 20 pence when bought in bulk), and which contrasted the ‘lies’ told by Parliamentarians with the ‘figures’ and ‘facts’ provided by LHE.^93^ Some key themes emerged in the research of LHE, demonstrative that tensions remained particularly in terms of mediating between regional and local anxieties while running a national campaign.

Notably, through multiple reports in the late 1980s and 1990s, the group was keen to challenge the so-called ‘trendy’ idea that London had relatively more hospital beds than the rest of the country.^94^ A figure regularly cited by newspapers in this period, and which has been supported by Rudolf Klein since, was that London’s hospital and community services received 20% of the NHS’s overall budget, despite only representing 15% of the population.^95^*Health Emergency* pinpointed seven ‘erroneous assumptions’ within this figure, using Ministry of Health and Department of Environment data, in particular in terms of recognising higher deprivation in London, existing bed cuts, and how populations from across the country used London’s healthcare systems.^96^

In this use of information, LHE’s work was akin to that of large and professionalised non-governmental organisations in this period, producing reports and undertaking detailed analysis. Notably, however, and again reflecting the small size of this group, LHE staff were not afraid to explicitly challenge the quality of research or ‘phony figures’ produced by other bodies, in colourful terms.^97^ While describing the King’s Fund as ‘impeccably inoffensive’ in general, *Health Emergency* featured the think-tank’s 1992 report into London’s hospitals in its ‘crackpots corner’, criticising its ‘poor quality research’.^98^ Another report on London’s hospitals also released in 1992, the Tomlinson Report, was criticised in *Health Emergency* as ‘deliberately and selectively’ ignoring certain statistical information, again about the uniqueness of London.^99^ LHE contended that the authors of this report had not been objective at all, but rather had sought merely to provide a ‘fig-leaf of academic respectability’ for the pre-existing government preferences: ‘massive cutbacks in hospital services’.^100^ From the 1980s therefore, LHE was constructing itself a role in judging, creating and analysing the validity, importance and accuracy of new bodies of information.

### Information, experience and emotion as expertise

Claims to be providing information and analysis became increasingly significant in this period. LHE felt that it was effective in this endeavour—its annual report from 1985 heralded as one of the ‘clearest indicators’ of its own growing influence ‘the rather clumsy effort’ of Thames Regional General Managers to establish a ‘joint propaganda unit on London … to combat our exposure of cuts and plunging standards in the capital’.^101^ The information provided by LHE was, at the time, taken seriously as a potential threat by the Department of Health and Social Security. In 1985, the *Guardian* circulated a private memorandum from the Department stating that the media focus on ‘cuts and the threat to London’s health services’ was ‘fuelled by analyses published by the GLC and the various health emergency groups’.^102^

The provision and construction of information was significant, and was presented as a mode through which to offer ‘neutral’ and ‘objective’ analysis about the NHS. Yet the formation and use of this information was never far removed from broader political and partisan debate. While LHE criticised information provided by other groups, in turn, the information provided by LHE was challenged by right-wing commentators. Speaking in 1994, Patrick Jenkin, former Health Minister in Thatcher’s government, for example, derided this group and others as ‘professional knockers’ producing ‘knocking copy’.^103^ LHE was labelled as ‘militant’, ‘extremist’ and ‘secretive’ by the Conservative Medical Society in 1989, and as part of the ‘hard left’ by the *Daily Mail* in 2002.^104^ Looking back retrospectively in a witness seminar of 2010, retired civil servants and politicians dismissed ‘Save Our Hospital’ campaigns of the early-1990s in derogatory terms, as an inevitable response to change, and buoyed solely by media interest.^105^ The creation of information was, of course, political, and did not create ‘objective’ new forms of expertise, nor entirely evade the broader politics of welfare state debate.

While information was being prized in the 1980s, and used to defend against criticism of ‘politicisation’, it is also important to look at what types of expertise campaigners were not using. Notably in this decade, there was less use by NHS campaigners of the politics of personal experience and emotion. This is perhaps surprising, given the personal nature of healthcare and concurrent media interest in disease as an emotional construction over this period.^106^ More broadly, an expertise of experience was emergent over these decades for example in New Social Movements, and also in small voluntary groups promoting the rights of carers, drug addicts, disabled people and parents.^107^ These groups all emphasised, in various ways, that the personal was political, and that their personal experiences had given them forms of expertise, and the right to be consulted and heard on the public stage. In contrast to these movements, and in the context of hospital closures, however, LHE staff did not tend to use personal stories to illustrate the effects of cuts.

When the organisation did discuss individual cases, this was primarily in terms of explaining the causes of individuals who had been unfairly victimised at work, or to criticise the unrepresentativeness of policy elites. In terms of the latter, *Health Emergency*’s regular column ‘Top R(H)AT’ satirised members of Regional Health Authorities, for example for having ‘learnt to represent the people of London by going to a typical London school, Eton’, or living in ‘but a small town house compared to the family home’.^108^ When describing Health Secretary Bottomley, *Health Emergency* argued that it was surprising that despite having worked as a psychiatric social worker, Bottomley showed no ‘greater sympathy for the plight of the mentally ill than her ill-starred predecessor’.^109^ More broadly, however, LHE attacks against Bottomley were in terms of her allegedly poor grasp of facts and figures about the NHS, embedded within the satirical claim that: ‘Don’t bother her with the facts: Bottomley just wants an excuse to close 4,200 beds’.^110^

NHS campaigning was thus notably distinct from the broader campaigning of many small voluntary groups in the 1980s, which drew on experiential and emotional forms of expertise. This reflected the significance of individual voluntary organisations in shaping NHS activism. For groups such as LHE and NHS Unlimited, leaders were particularly interested in analysis and information, rather than in using personal narratives as campaigning tools. The groups were also constructing an activism which surrounded information and was designed to transcend individual stories. In part, the focus on information rather than experience also reflected a growing sense in which the NHS was perceived as ‘special’ in public policy debate over this period, with growing recognition across substantial NHS reforms about the sheer ‘bigness’ of this institution. While individual voluntary groups could claim to hold knowledge from experience of specific institutions or diseases, the size of the NHS evaded the construction of a specific expert public group. In this context, it was the NHS itself which would become responsible for collecting public opinions, using large-scale mechanisms to capture patient testimony—‘Public and Patient Involvement’ tools—rather than relying on external information provided by voluntary groups or communities.^111^

The specific shape of this activism—focusing on information rather than personal experiences—also reflected and shaped a context in which much media represented the expression of emotion by NHS campaigners, and particularly by female campaigners, as uninformed and irrational.^112^ For example, in the context of the proposed closure of the Elsie Inglis maternity hospital in Edinburgh in the mid-1980s, hospital management wrote to local press to position the concerns of women as in opposition to new technologies and established research practice. Letters emphasised also that the emotional activism of these women would itself cause and spread negative emotions: annoyance to local staff and irritation and anxiety for mothers and expectant mothers.^113^ The representation of emotion was used to undermine the position of women in these debates, with narratives about modernity, technology and information being privileged.

The 1980s therefore saw the inception of a period in which comprehensive, detailed and prepared information was being used, contested and exchanged by and between pressure groups, health staff and central government, all hoping to shape the development of the NHS in a period in which its future felt increasingly uncertain. LHE’s emphasis on information-based campaigning was a purposeful and tactical construct, particularly used in a context where the expression of emotional and experiential campaigning, important in other fields, was used to devalue lobbying around the NHS. The dismissal of personal perspectives in campaigning at this point was, again, in part a response to a shift from local to national perspectives, but also a reflection of a politicised context in which discussions of facts and figures could be shrouded as ‘neutral’. While building on work from the 1960s and 1970s, information-based campaigning—especially as constructed to work ‘above’, alongside or against Thatcherism, while utilising government leaks—was distinct to the NHS in the 1980s, and part of a new mode of campaigning culture.

## Conclusion

In the post-war period, the roles and responsibilities of the state were extended through new welfare provisions, a more comprehensive education system and the inception of the NHS. New forms of voluntary action emerged around health and well-being, including campaigns about patient inclusion, NHS staff unrest and against the closure of local, community hospitals. Activism was often founded on local areas, through Community Health Councils or by campaigns which hailed individual hospitals as ‘modern’, ‘beautiful’, ‘small’ or ‘cosy’. Campaigning around the NHS changed significantly in the 1980s. As the post-war consensus and welfare system came under challenge or ‘crisis’, Margaret Thatcher’s administration sought to make cuts to the NHS and social services.^114^ In response to a perceived threat to the NHS as a whole, and capitalising on new forms of left-wing engagement, new types of activism developed. Campaigning explicitly in defence of the NHS, as a whole, was forged in the 1980s, and actively promoted by groups such as LHE.

These ‘new times’ for NHS campaigners were not solely defined by an increasing association with left-wing politics, and the 1980s also saw the development of new modes of political engagement.^115^ The 1960s and 1970s activism around hospital closures was dominated by physical and active protest, such as marches, sit-ins and occupations. In the 1980s, by contrast, a new form of ‘information-based’ activism and expertise also developed: reflecting the production of more ‘information’ in the NHS; shifts towards ‘evidence-based medicine’ and ‘evidence-based policy’; and interests of small voluntary groups, and their attempts to stand ‘above’, or at a distance from, political divisions.^116^ Media relationships with voluntary groups shifted in this decade also: from documenting protests outside hospitals towards also republishing ‘leaks’ and ‘facts’ collated or created by campaigners.

Importantly, furthermore, the call to ‘Save Our NHS’, as a whole, as a national institution, dates primarily to the 1980s. The NHS was not always nor instantly prized, but rather was ‘learnt’ by the public.^117^ A call to ‘Save Our NHS’ was not merely one to focus on the national, rather than the local, landscape of healthcare providers, but also a call entwined with demands for the NHS to act equitably, and a call interlaced with a new vision of the NHS as embodying a set of values. Campaigners sought to make adherence to those ‘values’ an identity, to be expressed through material culture and activism. Thus, this analysis demonstrates that narratives about public attachment to the NHS must be interrogated, and not assumed as collective nor ‘national’ knowledge. NHS activism has been made and remade over time, following the conscious efforts of campaigners, often from small but significant voluntary groups. Studying these small voluntary groups, indeed, is critical to understanding how cultural and political visions of the NHS have emerged and been enacted, realised and rejected in daily life. The politics of the NHS cannot be understood solely through ‘top-down’ histories of ‘the politics of the service’, which dominated the historiography until 2008.^118^

Attempts to cast activism around the NHS in new terms have continued in recent years, facilitated by oral histories, witness seminars and reflexive personal accounts. Reassessments are visible for example in retrospective accounts by Conservative politicians looking to undermine the significance of the 1980s NHS activism, but equally also in the reflections from campaigners, who seek to pull focus from the tensions between campaigning on the local and national levels over the same period. A key challenge for contemporary British historians is in terms of how best to utilise such contemporary recollections, for example to provoke reflection or to direct us to new archival sources. Looking to these sources, indeed, demonstrates the extent to which an emotional and cultural politics of the NHS, established in the 1980s and reflected and in part negotiated by small voluntary groups, continues to be significant today.

The idea of the NHS as a national system, embedded with unique meaning, was visible in the Olympics Opening Ceremony of 2012 and in the celebrations of the 70th Anniversary of the NHS in July 2018. The ways in which politicians have used the NHS to garner public favour remained clear in the calls around the EU Referendum to provide £350 million a week more for the service. Campaigners continue to call for ‘information-led’ expertise—initiating judicial reviews and freedom of information requests around the NHS, and producing significant reports. This type of campaigning is now positioned as against ‘fake news’ and the ‘post-truth society’. Activism in defence of ‘the NHS’, therefore, as developed during the 1980s, has spread beyond its initial foundation in left-wing and anti-Thatcherite politics and now inflects, reshapes and looks to critique our contemporary understandings of NHS policy and history.

## Notes

1. As quoted in, Wagg, *The London Olympics of 2012: Politics, Promises and Legacy*, 82–83.

2. As quoted in, Alexandra Topping and Rachel Williams, “Opening Ceremony Dancers: ‘It Truly Was a Phenomenal Spectacle,” *Guardian*, 28 July 2012.

3. See note 1 above.

4. “Olympics Opening Ceremony: US Media Reacts to ‘Peculiar’ British Festival,” *Guardian*, 28 July 2012.

5. See note 2 above.

6. Ben Glaze, “London Olympics Opening Ceremony Celebrating NHS Faced Being Cut due to ‘Political Pressure’,” *Daily Mirror*, 10 July 2016.

7. Figures from 1985 until 1994, when accounts are available (stored in the Modern Records Centre, Coventry, hereafter MRC, 1112/D/1/1-8, *Annual Reports 1985–1994*.)

8. See excellent summary of these historiographical debates in: Gorsky, “The British National Health Service,” 441–443.

9. Stewart, *The Battle for Health*

10. Seaton, “Against the ‘Sacred Cow’,” 426–427.

11. Gorsky, “The British National Health Service,” 442.

12. Gorsky and Preston, “The Resource Allocation Working Party,” 12.

13. See for example: Thomson, *The Problem of Mental Deficiency*; Milward, “A Disability Act?”; Milward, “Expertise, Disability and the Government,” 1965–1977”; Sewell, “Family Carer in Britain”; Moore, “Food As Medicine”; Bivins, *Contagious Communities*; Berridge, *AIDS in the UK*; Mold and Berridge, *Voluntary Action and Illegal Drugs*; King, *Family Men* and Davis, *Modern Motherhood*.

14. Rivett, *The Development of the London Hospital System*. See also: Gorsky, “The British National Health Service,” 444 and Edgerton, “C. P. Snow as Anti-Historian of British Science,” 187–208.

15. Ministry of Health, *A Hospital Plan for England and Wales*, 1962; Ministry of Health, *The Hospital Building Programme*, 1966.

16. Gorsky and Preston, “The Resource Allocation Working Party,” 13.

17. Gorsky, and Millward, “Resource Allocation for Equity in the British National Health Service,” 69–108.

18. See note 16 above.

19. Begley, Sheard, and Mackillop, “The 1974 NHS Reorganisation.”

20. Harrison, “The Closed Shop,” 67, 79; Klein, *The New Politics of the NHS*, 80–81.

21. See: Mold, *Making the Patient-Consumer*; Crane, “History of Public Consultation Matters.”

22. Hutt, Heath and Neighbour, *Confronting an Ill Society*, 50; Widgery, *The National Health Service*, 166.

23. Mold, “Patient-Consumer in Britain”; Faulkner, “User Involvement in 21st C”; Tait and Lester, “Encouraging User Involvement”; Crossley and Crossley, “Patient Voices” and Jones, “What Does a Hospital Mean?,” 254–256.

24. “In Defence of a Place of their Own,” *Guardian*, 28 February 1977, 9.

25. MRC, MSS.292D/847.3/4, “Hands Off Rhydlafar,” 1.

26. “Women’s Hospital Losing Survival Battle,” *Guardian*, 25 April 1978, 26; Robin Laurance, “Stretcher Case”, *Guardian*, 8 December 1974, 9.

27. Moon, and Brown, “Closing Barts,” 53; “New Move to Save Hospital,” *Observer*, 30 May 1971, 2. See also, an earlier example: “Final Attempt to Save Hospital,” *Manchester Guardian*, 29 September 1939, 2.

28. “Staff Tries to Save Hospital,” *Guardian*, 24 December 1973, 4.

29. Malcolm Pithers, “Thousands Lobby Vaughan in Attempt to Save Hospital,” *Guardian*, 8 August 1979, 3.

30. “New Move to Save Hospital,” *Observer*, 30 May 1971, 2

31. Crook, “The Women’s Liberation Movement, Activism and Therapy”; Crane, *Child Protection in England*, Chapter 6: ‘Mothers, Media, and Individualism in Public Policy’.

32. “A Tribute for Helping to Save Hospital,” *Guardian*, 24 February 1965, 20.

33. Bethnal Green Hospital Campaign 1977–1978.

34. See note 24 above.

35. Ibid.

36. Ibid.

37. Jones, “What Does a Hospital Mean?,” 254–256.

38. “The Day that ‘Mini-Thuggery’ of Old People Became a Case of Murder,” *Guardian*, 20 May 1978, 11.

39. Margaret Thatcher, “Speech to Conservative Party Conference,” 10 October 1986, https://www.margaretthatcher.org/document/106498.

40. MRC, London Health Emergency, *Annual Reports* and *Annual Accounts*.

41. See accounts in Modern Records Centre, 1112/D/1/1–8, *Annual Reports 1985–1994*.

42. Gorsky, “The British National Health Service,” 445–456. See also: Gamble, *The Free Economy and the Strong State*; Pierson, *Dismantling the Welfare State?*

43. Gorsky, “The British National Health Service,” 446. See also: Gamble, *The Free Economy and the Strong State*; Pierson, *Dismantling the Welfare State?*

44. Payling, “Socialist Republic of South Yorkshire,” 602–627; Brooke, “Space, Emotions and the Everyday,” 1–33.

45. Brooke, “Space, Emotions and the Everyday,” 8, 11, 13.

46. John Lister interviewed by Jennifer Crane, Whitnash, 4 July 2016.

47. MRC, 1112/D/1/1, *Annual Report, March 1986–March 1987*, 2.

48. Dobson, “Foreword”; Lister, *Cutting the Lifeline*, 10.

49. Lister, *Cutting the Lifeline*, 11.

50. “Camberwell,” *Health Emergency*, April 1984, 3; “Lewisham and Southwark,” *Health Emergency*, April 1984, 4.

51. “Oxford,” *Health Emergency*, February 1988, No. 18, 5.

52. “The Battle to Save Thornton View,” *Health Emergency*, April 1984, 7; “Support the Miners,” *Health Emergency*, April 1984, 8.

53. “Miners’ Fight Is Our Fight,” *Health Emergency*, June 1984: 7.

54. “LHE 2016: A Potted H,” LHE website; Posters in: MRC, London Health Emergency, Campaign Materials, 1112/P/5/2.

55. MRC, “Hands Off Our Health Service,” 3.

56. “Clarke’s Bill Heads for Fiasco,” *Health Emergency*, July 1990, No. 25, 8; “Vanishing Nurses,” *Health Emergency*, No. 19, May 1988, 2.

57. “London: The Argument Is Won, but the Fight Goes on,” *Health Emergency*, August 1994, 4; “The Hospital that Was Murdered,” *Health Emergency*, November 1997, 4.

58. MRC, MSS.292D/847.3/4, *National Health Service* (*NHS*): Hospitals, 1978–1981, 847.3, Fightback, *Health—Not Cuts* flyer, 3.

59. *Health Emergency*, May 1991, 1.

60. Advert, *Health Emergency*, July 1990, 5.

61. Health Emergency, No. 63, January 2007, “Help us Step Up Our Campaigning Work!,” 8.

62. See for example: “Making It Really OUR Health Service,” *Health Emergency*, June 1984, 5; “Drug Cuts Hit Kids and OAPs,” *Health Emergency*, January–February 1985, 3; Sue Lister, “Cancer and the Cuts”, *Health Emergency*, April 1984, 2; “The Fraud of ‘Community Care’,” *Health Emergency*, April 1984, 2.

63. Berridge, Virginia, “The Black Report and *The Health Divide*,” 2003, http://history.lshtm.ac.uk/2016/06/13/the-black-report-and-the-health-divide-black-report/ (8 August 2018).

64. MRC, MSS.292D/847.3/4, *National Health Service* (*NHS*): Hospitals, 1978–1981, 847.3, Socialist Medical Association, *Socialism & Health*, 1.

65. See for example: Sue Lister, “Cancer and the Cuts,” *Health Emergency*, April 1984, 2; “Women Bear Brunt: The Fraud of ‘Community Care,” *Health Emergency*, April 1984, 2.

66. “Racist Priorities of the Medical Hierarchy,” *Health Emergency*, April 1984, 6.

67. Survey compiled through Surveymonkey from 23 March 2016–March 2017 (at https://www.surveymonkey.co.uk/r/QGWQW88).

68. MRC, London Health Emergency, *Annual Report, 1987–1988*, 5.

69. See note 46 above.

70. “What They Say about Us,” *Health Emergency*, June 1984, 6.

71. King’s, *London Health Care 2010*; Tomlinson, *London’s Health Service.*

72. “Less Beds per Head,” *Independent*, 1 June 1994, 6

73. Lister, “London Says No!,” *Health Emergency*, January 1993, 1.

74. Crane, “Buggy Armies.”

75. Klein, *The New Politics of the NHS*, 216.

76. Foster and Gledhill, “Left Wingers Accused of Driving Nurses to Strike,” *The Times*, 29 January 1988, 2.

77. See note 46 above.

78. MRC, London Health Emergency, *Annual Report 1985*, 1–2.

79. MRC, London Health Emergency, *Annual Report March 1986–March 1987*, 1.

80. Norton-Taylor and Hencke, “DHSS Has a Word about Words,” *Guardian*, 13 December 1985, 4; “Diary,” *Guardian*, 23 December 1987, 26; Foster and Gledhill, “Left Wingers Accused of Driving Nurses to Strike,” *The Times*, 29 January 1988, 2; “Cuts in London,” *Lancet*, 23 January 1988, 331, 8579, 195–196; “London’s Health Services,” *Lancet*, 7 May 1988, 331, 8593, 1062–1064; Mintowt-Czyz, “Health Boss who Spent £104,000 on New Office,” *Daily Mail*, 5 July 2000, 29 and McSmith and Mills, “Winter Looms over New NHS Funding,” *Guardian*, 7 December 1997, 5.

81. Thane and Davidson, *The Child Poverty Action Group, 1965 to 2015*.

82. Ham, “Community Health Council Participation,” 227.

83. Klein, *The New Politics of the NHS*, 213; Mold, “Patient-Consumer in Britain,” 514 and Mold, “The Art of Medicine,” 1287.

84. Bodleian Library, Oxford, 1514 d. 132, *Going Private: The Case against Private Medicine*, 1982, inside of back cover.

85. Wellcome Archives, SA/PHG, “Women’s Group Receives £7000,” 3

86. See: Hilton, McKay, Crowson, Mouhot, *The Politics of Expertise*; Crowson, McKay, Hilton, *NGOs in Contemporary Britain*; Hilton, Crowson, Mouhot, McKay, *A Historical Guide to NGOs* and Hilton, “Politics Is Ordinary.”

87. “Countdown to Closures,” “Merger Mania,” *Health Emergency*, June 1992, 2.

88. Norton-Taylor and Hencke, “DHSS Has a Word about Words,” *Guardian*, 13 December 1985, 4

89. See above 45, 2016.

90. Ibid.

91. LHE, *Annual Report 1985*, 5; “Act Now to Save the NHS!,” *Health Emergency*, November 1993, 3 and “Countdown to Closures,” *Health Emergency*, June 1992, 1.

92. Vincent, *The Culture of Secrecy*, 269.

93. MRC, London Health Emergency, Campaign Materials, 1112/P/5/2, London’s Hospitals: Under Attack, 1.

94. “The Myth of London’s ‘Surplus’ Hospital Beds,” *Health Emergency*, February 1992, 4.

95. Klein, *The New Politics of the NHS*, 207.

96. “The Myth of London’s ‘Surplus’,” 4.

97. “Phony Figures,” *Health Emergency, Health Emergency*, June 1984, 3.

98. “Crackpots’ Corner,” *Health Emergency*, September 1992, 4.

99. “Seven Things the Prof Got Wrong,” *Health Emergency*, January 1993, 5.

100. Lister, “Inquiry Sharpens Hospital Axe,” *Health Emergency*, February 1992, 1.

101. MRC, London Health Emergency, *Annual Report 1985*, 1.

102. See note 88 above.

103. Hansard, House of Lords, 12 January 1994, vol. 551, cc. 146.

104. Conservative Medical Society comments as reported in: Martin, “The Sharp End,” *Health Emergency*, October 1989, 4; MicKinstry, “March of the Hard-Left,” *Daily Mail*, 21 January 2002, 30–31.

105. *Tomlinson Report and After*, 40–41.

106. Interesting on the relations between media and disease, particularly heart transplants: Nathoo, *Hearts Exposed.*

107. Klein, *The New Politics of the NHS*, 105–106; Crane, “Painful Times,” 450–476. On New Social Movements see Lent, *British Social Movements since 1945*; Meyer and Tarrow, *The Social Movement Society*; Tarrow, *Power in Movement* and Crossley, *Making Sense of Social Movements*.

108. “Top R(H)AT,” *Health Emergency*, April 1984, 5.

109. Bulletin of Hands Off Our NHS, “Bottomley’s Bed Blitz?,” *Health Emergency*, June 1992, 1.

110. See note 99 above.

111. See note 21 above.

112. “The fight for Elsie’s,” *Edinburgh Evening News*, 18 December 1986, 4

113. “A clean bill of health at the Eastern”; Opposing these arguments was a member of Edinburgh Health Council in: ‘Medical retreat into irrationality’.

114. Robinson, Schofield, Sutcliffe-Braithwaite, and Thomlinson, “Telling Stories about Post-War Britain.”

115. Campsie, “Socialism Will Never Be the Same Again,” 166–188.

116. On the construction of evidence-based medicine and policy in this period see for example, Sackett, “Evidence-Based Medicine,” 3–5; Davidoff, Haynes, Sackett and Smith, “Evidence Based Medicine,” 1085–1086 and Muir Gray, “Evidence Based Policy Making,” 988–989.

117. Thomson, “The NHS and the Public.”

118. Tendency observed in: Gorsky, “The British National Health Service,” 438. This historiographical tendency has been remarkably reversed in recent years, notably, and in a variety of ways, by a series of Wellcome Trust Senior Investigator Award projects studying the ‘Cultural History of the NHS’ at the University of Warwick, ‘Governance of Health’ at the University of Liverpool, ‘Placing the Public in Public Health’ at the London School of Hygiene and Tropical Medicine, and—bringing a global context—‘Health Systems in History: Ideas, Comparisons, Policies’ at the London School of Hygiene and Tropical Medicine.
